# Effect of Green Tea Supplementation on Antioxidant Status in Adults: A Systematic Review and Meta-Analysis of Randomized Clinical Trials

**DOI:** 10.3390/antiox10111731

**Published:** 2021-10-29

**Authors:** Niloufar Rasaei, Omid Asbaghi, Mahsa Samadi, Leila Setayesh, Reza Bagheri, Fatemeh Gholami, Neda Soveid, Krista Casazza, Alexei Wong, Katsuhiko Suzuki, Khadijeh Mirzaei

**Affiliations:** 1 Department of Community Nutrition, School of Nutritional Sciences and Dietetics, Tehran University of Medical Sciences, Tehran 14176-13151, Iran; n.rasaei71@gmail.com (N.R.); mahsa_samadi@ymail.com (M.S.); setayesh.leila@yahoo.com (L.S.); gholami_fghlm67@yahoo.com (F.G.); neda.soveid@gmail.com (N.S.); 2 Cancer Research Center, Shahid Beheshti University of Medical Sciences, Tehran 14167-53955, Iran; omid.asbaghi@gmail.com; 3 Department of Exercise Physiology, University of Isfahan, Isfahan 81746-73441, Iran; will.fivb@yahoo.com; 4 Marieb College of Health and Human Services, Florida Gulf Coast University, Fort Myers, FL 33965, USA; krista.casazza@gmail.com; 5 Department of Health and Human Performance, Marymount University, Arlington, VA 22207, USA; awong@marymount.edu; 6 Faculty of Sport Sciences, Waseda University, 2-579-15 Mikajima, Tokorozawa 359-1192, Japan

**Keywords:** green tea, oxidative stress, total antioxidant capacity, malondialdehyde

## Abstract

It is well-established that green tea supplementation has antioxidant properties. However, whether green tea supplementation leads to oxidative stress reduction remains unclear, as clinical investigations on this subject have yielded inconsistent outcomes. Consequently, we aimed to determine the effects of green tea supplementation on oxidative stress in adults. A systematic search of English language publications up to 21 August 2021 was carried out in PubMed, Scopus, Embase, and ISI Web of Science, utilizing pertinent keywords. These searches included randomized controlled trials (RCTs) evaluating the relationship between green tea supplementation, malondialdehyde (MDA), and total antioxidant capacity (TAC) in adults. A random-effects model was used to estimate the weighted mean difference (WMD) and 95% confidence intervals (95% CI). Meta-regression and non-linear dose-response analyses were performed to investigate the association between the dosage of green tea (mg/day) and the duration of the intervention (weeks) with pooled effect size. Sixteen RCTs with seventeen arms including 760 participants met the inclusion criteria. Our results indicated that green tea supplementation had significant effects on TAC (weighted mean difference [WMD]: 0.20 mmol/L; 95% CI: 0.09, 0.30, *p* < 0.001) and significant heterogeneity between studies (I^2^ = 98.6%, *p* < 0.001), which was largely related to gender and body mass index (BMI). Subgroup analysis in TAC identified a significant relationship except with low dose supplementation and obese individuals. No relationship between MDA and green tea supplementation was observed in any subgroups; however, meta-regression analysis revealed a linear inverse association between the dosage and significant change in MDA (*r* = −2117.18, *p* = 0.017). Our outcomes suggest that green tea supplementation improves TAC and affects MDA based on the dose of the intervention in adults. Future RCTs with longer durations are needed to expand our findings.

## 1. Introduction

All metabolic processes generate reactive oxygen species (ROS), which can induce oxidative damage to tissue and cell constituents such as lipids, proteins, and nucleic acids [1,2] when left unbalanced. Indeed, the overproduction and accumulation of large amounts of ROS lead to oxidative stress [3,4,5], which represents a major contributor to the risk, onset, and progression of chronic diseases [6,7,8,9,10,11,12]. A key component of oxidative stress implicated in adverse health effects is lipid peroxidation of polyunsaturated fatty acids and the subsequent production of malondialdehyde (MDA) (a major indicator of lipid oxidation) [9]. Therefore, interventions to intensify the body’s antioxidant response to counteract oxidative stress and MDA production are desperately needed. 

Natural dietary polyphenolic compounds are potent antioxidants that decrease oxidative stress and protect the body against several oxidative stress-associated diseases [13,14]. Among the common sources of natural dietary polyphenol in foods and beverages is green tea (originate from the leaves of Camellia sinensis), which has high antioxidant properties related to various compounds such as phenolics and flavonoids, and flavonols [15,16,17]. In fact, the major polyphenols with antioxidant properties in green tea are epicatechin (EC), epigallocatechin-3-gallate (EGCG), epicatechin-3-gallate (ECG), epigallocatechin (EGC), and gallocatechin gallate (GCG), which are also called catechins [18]. While some studies have reported that EGCG and other catechins in green tea increase the activity of antioxidant enzymes, decrease indices of oxidative damage and prevent and treat some diseases [19,20,21], there are equivocal effects on markers of oxidative stress in diabetes, metabolic syndrome, β–thalassemia major, and obesity [9,13,22,23,24,25]. Methodological issues such as non-randomization, different outcome measures, short duration of treatment, low study quality, and population heterogeneity have precluded a comprehensive understanding of the extent to which green tea supplementation may exert a beneficial effect on oxidative stress. Further, the potential effectiveness of green tea supplementation in mitigating oxidative stress among adults with pre-existing inflammatory conditions has not been clarified. Thus, we performed a systematic review and meta-analysis of randomized controlled trials (RCTs) to evaluate the relationship between green tea supplementation and oxidative stress markers, as assessed by the MDA and total antioxidant capacity (TAC), in adults with and without inflammatory health conditions.

## 2. Materials and Methods

Findings from this systematic review and meta-analysis were reported based on the Preferred Reporting Items of Systematic Reviews and Meta-Analysis (PRISMA) guideline [26]. The PICOS model was used to prepare the question: population (all individuals), intervention (green tea), comparison (studies which had control group), outcome (studies that reported two markers of oxidative stress including MDA and TAC), and study design (RCT, Registration code: CRD42021240026) [27]. 

### 2.1. Search Strategy

We conducted database searches of PubMed, Scopus, Embase, and ISI web of science from inception to 21 August 2021. All relevant studies written in the English language were included using. Medical subject headings (MeSH) and text words. Search words included: 

“green tea” OR “green tea extract” OR “green tea extract AR25” OR “catechin” OR “catechins” OR “EGCG” OR “Camellia sinensis” OR “tea polyphenols” OR “catechinic acid” OR “acid catechinic” OR “sinensis Camellia” OR “Thea sinensis” OR “sinensis Thea” OR “tea polyphenols” AND “oxidative stress” OR malondialdehyde OR MDA OR glutathione OR GSH OR “total antioxidant capacity” OR TAC OR “total antioxidant status” OR TAS AND.

intervention OR “intervention study” OR “intervention studies” OR “controlled trial” OR randomized OR randomized OR random OR randomly OR placebo OR assignment OR “clinical trial” OR trial OR assignment OR “randomized controlled trial” OR “randomized clinical trial” OR RCT OR blinded OR “double blind” OR “double blinded” OR trial OR “clinical trial” OR trials OR “pragmatic clinical trial” OR “cross-over studies” OR “cross-over” OR “cross-over study” OR parallel OR “parallel study” OR “parallel trial”. Reference lists of the included studies were manually checked to identify additional articles. Unpublished documents and grey literature such as thesis, patents, and conference papers were not included.

### 2.2. Eligibility Criteria

Studies were included if they met the following criteria: RCT design, population aged >18 years, and reported MDA and/or TAC in both the intervention and placebo groups. Studies with duplicated data, non-RCT design, animal studies, studies that assess the effects of green tea supplementation and other interventions, and those with insufficient data for the outcomes of interest or did not meet inclusion criteria of our meta-analysis were excluded.

### 2.3. Data Extraction

Two independent researchers extracted data and evaluated any disagreement was resolved through open discussion with a third independent investigator (KhM). First author’s name, year of publication, study location, duration and design, gender, mean age and mean body mass index (BMI) of population, the health status of participants, number of participants in each group, type and dose of green tea, and results (means and standard deviations for MDA and TAC before and after intervention) were extracted from the full text of included studies. 

### 2.4. Quality Assessment

Assessment of study quality each included study was precisely evaluated for quality by the Cochrane scoring system [28] of 7 points based on the following criteria: (1) random sequence generation, (2) allocation concealment, (3) blinding of participants and personnel, (4) blinding of outcome assessment, (5) incomplete outcome data, (6) selective reporting, (7) other biases. Three scores of L, H, and U could be given to each aforementioned item, which is interpreted as low risk, high risk, and unknown risk, respectively. Two studies had low random sequence generation [29,30]. Allocation concealment reported in 15 studies [4,5,10,13,23,29,30,31,32,33,34,35,36,37]. Moreover, 3 trials had a high risk of bias regarding blinding of participants, personnel [9,24,31], and 3 trials had low-risk outcome assessors [22,29,34]. Selective reporting was considered as low risk in one trial [10]. All of the studies showed a low risk of bias based on incomplete outcome data. Other sources of bias were low in 10 trials [5,10,13,22,29,30,32,34,35,37]. 

### 2.5. Statistical Analysis

This meta-analysis was performed using STATA software, version 14.0 (Stata Corp. LP, College Station, TX, USA). The effect of green tea supplementation on oxidative stress (MDA and TAC) was estimated by pooling mean and standard deviation (SD) values of the baseline and final of the studies in both intervention and placebo groups. Missing SDs for changes were calculated utilizing the following formula: SD change = square root [(SD baseline^2^ + SD final^2^) − (2 × R × SD baseline × SD final)], if the studies did not report mean and SD. We considered a correlation coefficient of 0.9 as the R-value of the mentioned formula [38]. We used the following formula to calculate standard errors (SE) from SD: SE√n. If a study provided medians (interquartile ranges), we converted them to means (SD) as described by Hozo and colleagues [39]. If the study results were reported graphically, we used GetData Graph Digitizer software to estimate mean (SD). At first, a fixed-effect model was performed to pool the data. A random-effects model was used to estimate the weighted mean difference (WMD) and 95% confidence (95% CI). Heterogeneity among studies was assessed with the Q test and the I^2^ index statistic. If *p* < 0.1 and I^2^ > 50%, it was considered that heterogeneity existed among studies. If *p* > 0.1 and I^2^ < 50%, fixed-effect models would be applied. Sensitive analysis and subgroup analysis were performed to evaluate the source of heterogeneity and verified the stability of results. In the sensitivity analysis, one study was omitted at each turn to evaluate the influence of each study on the results. Subgroup analysis was performed by stratifying study type, region, study quality score, and maximum category. Contour-enhanced funnel plots with an Egger linear regression test and a Begg rank correlation test were used to evaluate the potential publication bias. Meta-regression and non-linear dose-response analyses were performed to investigate the association between the dosage of green tea (mg/day) and the duration of the intervention (weeks) with pooled effect size. Because all the data used for analyses were extracted from the published studies, ethical approval and informed consent were not necessary. 

## 3. Results

### 3.1. Study Selection

**Figure 1:**
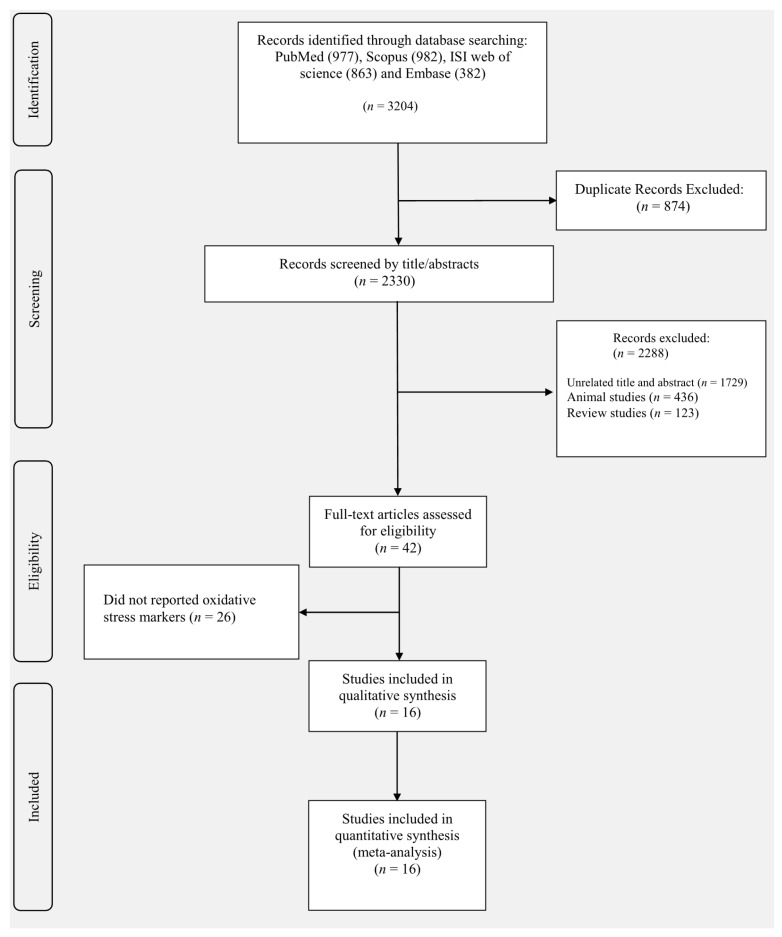
The flow chart of the literature search.

We found 3204 publications in Scopus, PubMed, Embase, and ISI web of science in the initial search. Of these, eight hundred seventy-four articles were duplicated. Thus, a total of 2330 articles were assessed for the title and abstract screening. After screening of title and abstract, 2288 unrelated studies were discarded due to primary evaluation of inclusion criteria: unrelated title (*n* = 1729), review (*n* = 123), and animal study (436). Consequently, 42 studies were retrieved for full-text review, 26 of which were excluded due to: lack of sufficient data and the use of two or more interventions at the same time [2,21,22,24,30,34,40,41,42,43,44,45,46,47,48,49,50,51,52,53,54,55,56,57,58,59,60,61]. Finally, sixteen RCTs with one double-arm study [4,5,9,10,13,22,24,29,37] were eligible for this systematic review and meta-analysis. The flow chart of the literature search is shown in Figure 1. 

### 3.2. Study Characteristics

Sixteen RCTs with one double-arm study assessing the effects of green tea supplementation on oxidative stress were identified. Included studies carried out in various countries such as Iran [5,9,22,24,33,36,37], Poland [4,29,31,32], Lithuania [13,31], Taiwan [34,35] and Brazil [10]. Publication dates ranged from 2009 and 2020. The follow-up period ranged from 2 weeks to 36 weeks. The sample size of the included studies ranged from eight to forty-eight participants. Some studies enrolled only females [35] or males [4,5,9,31,34,36], and the rest of included studies involved both genders [10,13,22,24,29,32,33,37], and one of them was unclear [31]. Type of intervention administration were green tea [9,22,24,34,35] and green tea extract [4,5,9,10,13,29,32,34,36,37]. Three studies were conducted on obese individuals [9,29,32], four studies on healthy individuals [4,5,31,34,36], five studies were conducted on diabetics [10,13,24,30,33,37], one study on β–thalassemia major [29] and one study on hypercholesterolemic participants [35]. The studies were performed in participants with different baseline BMI; five studies carried out in participants under 25 kg/m^2^ [4,5,22,31,34,36], eight studies over than 25 kg/m^2^ [9,10,24,29,30,32,33,35,37] and one study did not report BMI [13]. All studies were parallel RTCs [4,5,9,10,13,22,24,29,37]. The summary of the characteristics of the included studies is indicated in Table 1. Results from the quality assessment are shown in Table 2.

### 3.3. Meta-Analysis

#### 3.3.1. Effect of Green Tea Supplementation on TAC

**Figure 2:**
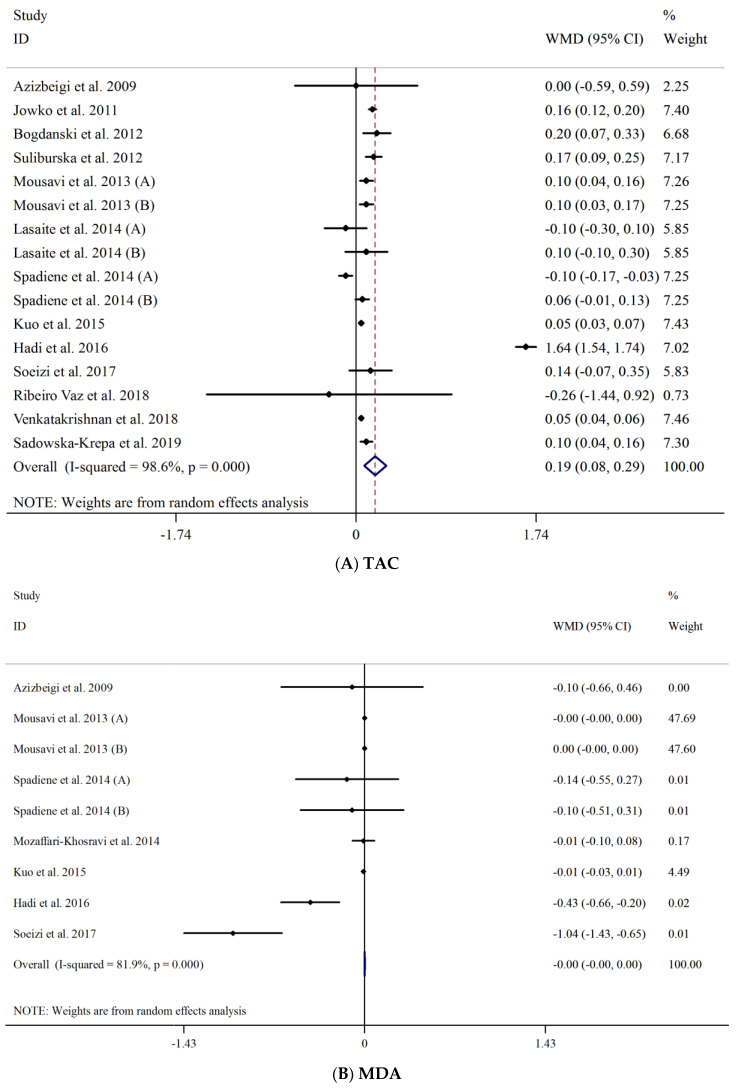
Forest plot detailing weighted mean difference and 95% confidence intervals (CIs) for the effect of green tea consumption on; (**A**) TAC; (**B**) MDA.

Overall, 16 studies with seventeen arms evaluated the effect of green tea supplementation on TAC. Pooled effect size from random effect model showed a significant increasing effect of green tea supplementation on TAC (WMD: 0.20 mmol/L; 95% CI: 0.09, 0.30, *p* < 0.001). There was significant heterogeneity between studies (I^2^ = 98.6%, *p* < 0.001) (Figure 2A). However, after subgroup analysis we observed significant effect of green tea supplementation on TAC in studies of less (WMD: 0.43 mmol/L; 95% CI: 0.10, 0.75, *p* = 0.010) or more than 8 weeks (WMD: 0.08 mmol/L; 95% CI: 0.02, 0.13, *p* = 0.004). Similar significant effects were also detected in green tea extract (WMD: 0.23 mmol/L; 95% CI: 0.03, 0.42, *p* = 0.019) and brewed green tea (WMD: 0.07 mmol/L; 95% CI: 0.03, 0.10, *p* < 0.001) and male (WMD: 0.38 mmol/L; 95% CI: 0.07, 0.69, *p* = 0.003), female (WMD: 0.05 mmol/L; 95% CI: 0.04, 0.05, *p* = 0.015) and both sexes (WMD: 0.12 mmol/L; 95% CI: 0.08, 0.17, *p* < 0.001). In addition, sub-group analysis based on dose showed that green tea supplementation for >400 mg/day had significant effect on TAC (WMD: 0.29 mmol/L; 95% CI: 0.09, 0.49, *p* = 0.004). In addition, subgroup analysis demonstrated that both healthy (WMD: 0.08 mmol/L; 95% CI: 0.02, 0.13, *p* = 0.004) and unhealthy status (WMD: 0.43 mmol/L; 95% CI: 0.10, 0.75, *p* = 0.010) significantly affect TAC. Sub-group analysis based on BMI showed that those with BMI = 18.5–24.9 and 25–29.9 had significant effect on TAC respectively (WMD: 0.38 mmol/L; 95% CI: 0.08, 0.68, *p* = 0.011) (WMD: 0.11 mmol/L; 95% CI: 0.07, 0.15, *p* < 0.001) (Table 3).

#### 3.3.2. Effect of Green Tea on MDA

Eight clinical trials evaluated the effect of green tea on MDA. Pooled effect size from random effect model showed a nonsignificant decreasing effect of green tea supplementation on MDA (WMD: −0.00 μmol/L; 95% CI, −0.00, 0.00, *p* = 0.636). There was significant heterogeneity between studies (I^2^ = 84.1%, *p* < 0.001) (Figure 2B). In addition, we performed subgroup analysis which did not indicate any changes in MDA following green tea supplementation; however, heterogeneity was reduced in interventions with less than 400 mg/day (I^2^ = 0.0%, *p* = 0.530), BMI = 25–29.9 (I^2^ = 0.0%, *p* = 0.609) and BMI ≥30 (I^2^ = 0.0%, *p* = 0.910) (Table 3). 

#### 3.3.3. Publication Bias and Sensitivity Analysis

**Figure 3:**
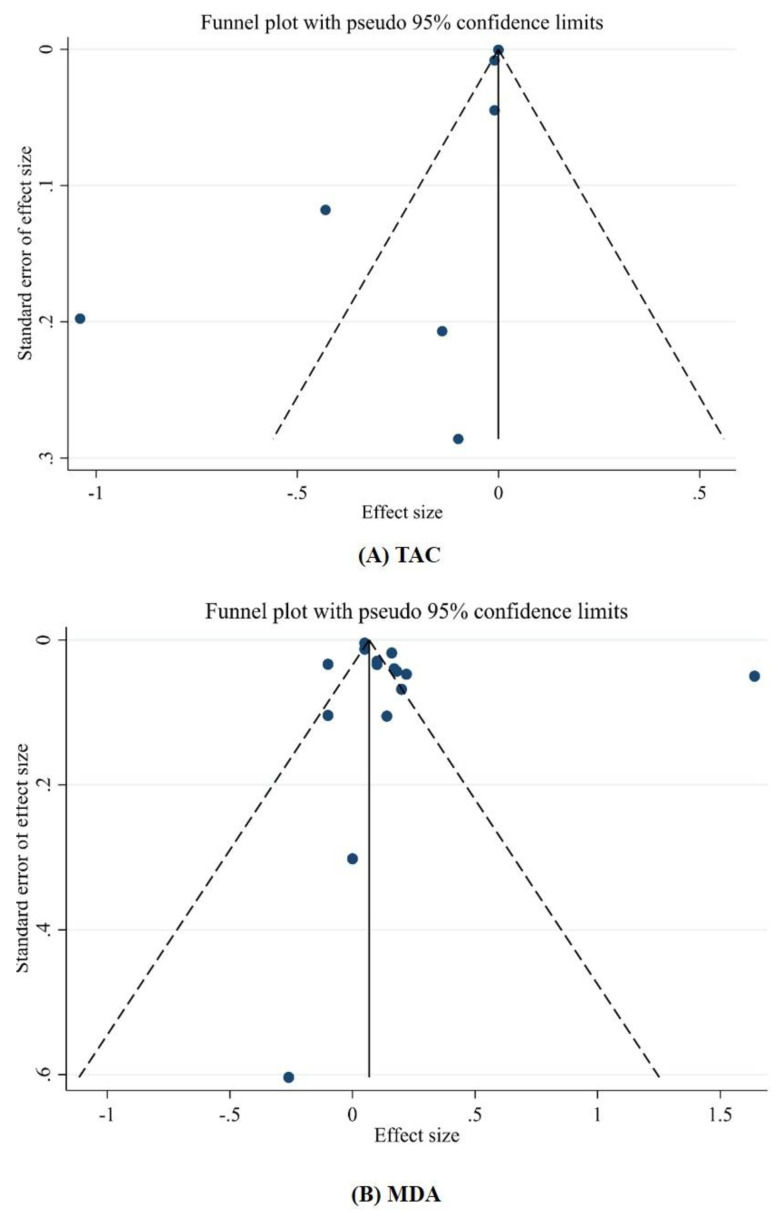
Funnel plot for the effect of green tea consumption on; (**A**) TAC; (**B**) MDA.

The sensitivity analysis indicated that removing any of the studies would not substantially change the effect of green tea supplementation on oxidative stress (Figure 2). We used Egger’s weighted regression tests to explore the publication bias. There was no evidence of publication bias for studies examining the effect of green tea supplementation on MDA (*p* = 0.335, Egger’s test) (*p* = 0.711 Begg test), and TAC (*p* = 0.093, Egger’s test) (*p* = 0.893 Begg test) (Figure 3A,B). 

#### 3.3.4. Non-Linear Dose-Response between Dose and Duration of Green Tea Supplementation and Antioxidant Status

**Figure 4:**
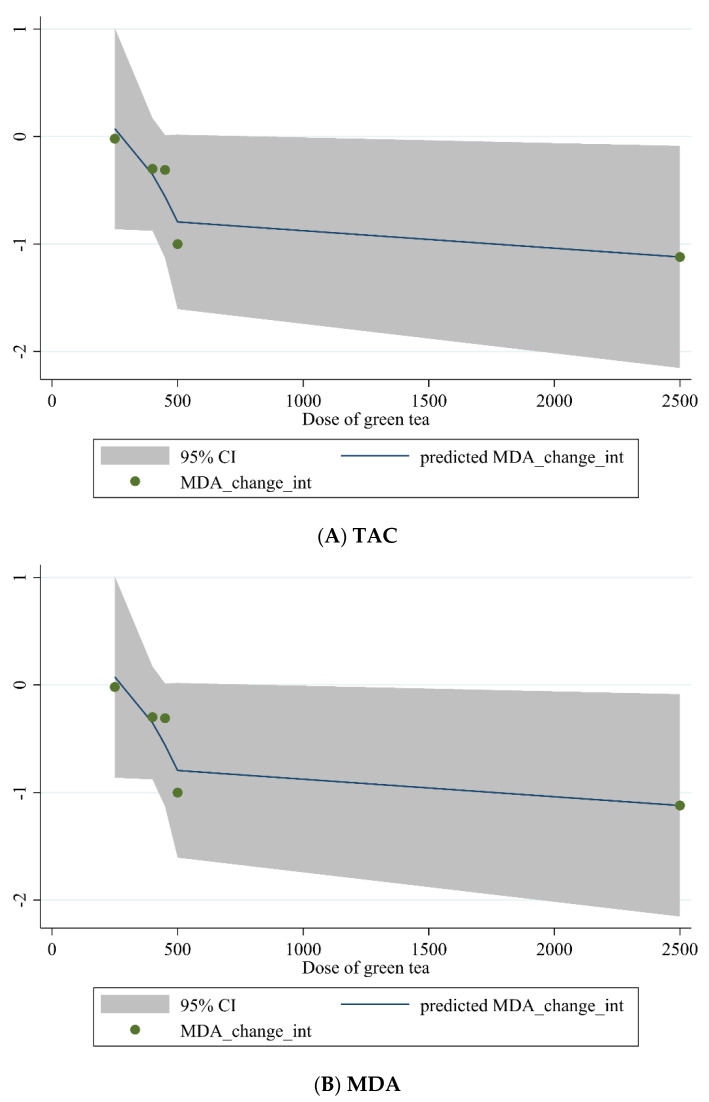
Non-linear dose-response relations between dose of green tea (mg/day) and predicted changes in (**A**) TAC and (**B**) MDA.

**Figure 5:**
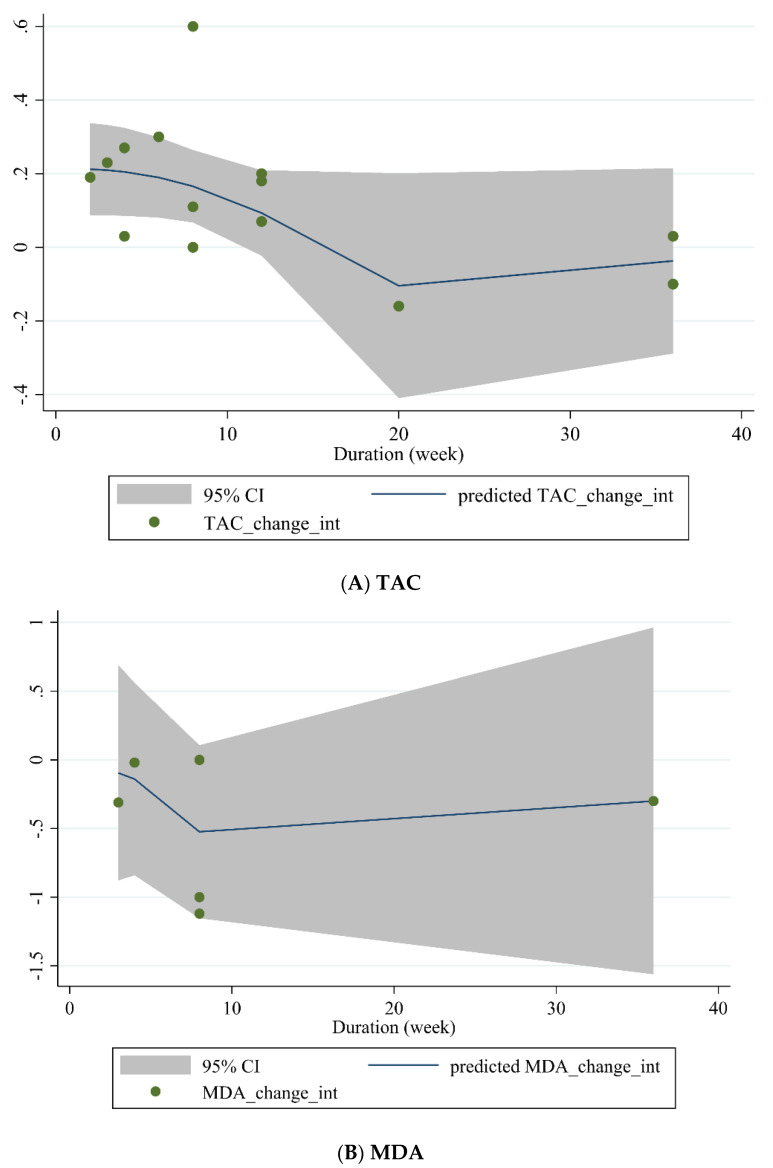
Non-linear dose-response relations between duration of intervention (weeks) and predicted changes in (**A**) TAC and (**B**) MDA.

Non-linear dose-response analysis was conducted between the dose and duration of green tea supplementation for TAC and MDA. Dose-response analysis indicated that green tea supplementation did not affect TAC and MDA concentrations (Figure 4A,B and Figure 5A,B).

#### 3.3.5. Meta-Regression Analysis

**Figure 6:**
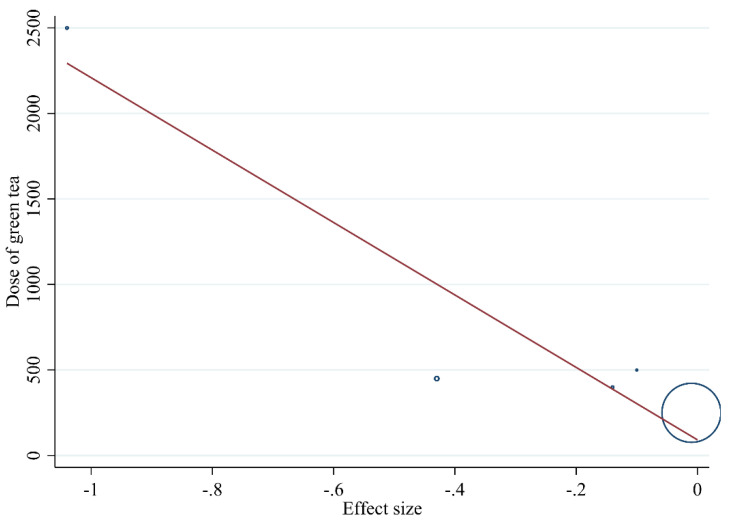
Random-effects meta-regression plot of the association between dose of green tea (mg/day) and MDA effect size.

**Figure 7:**
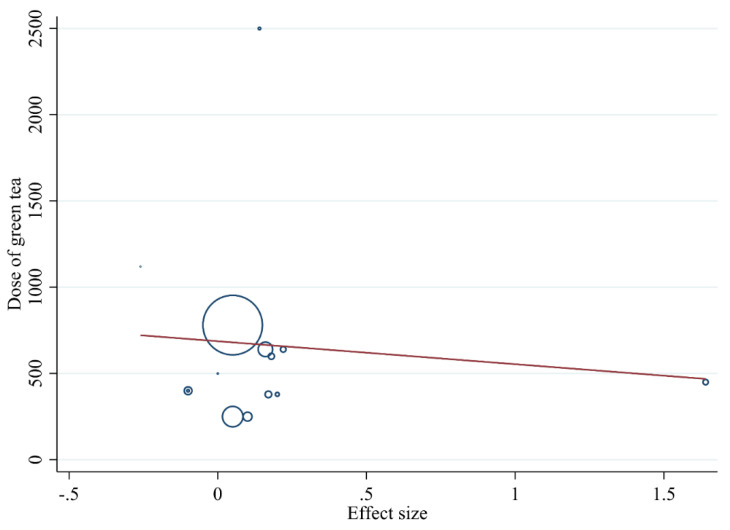
Random-effects meta-regression plot of the association between dose of green tea (mg/day) and TAC effect size.

**Figure 8:**
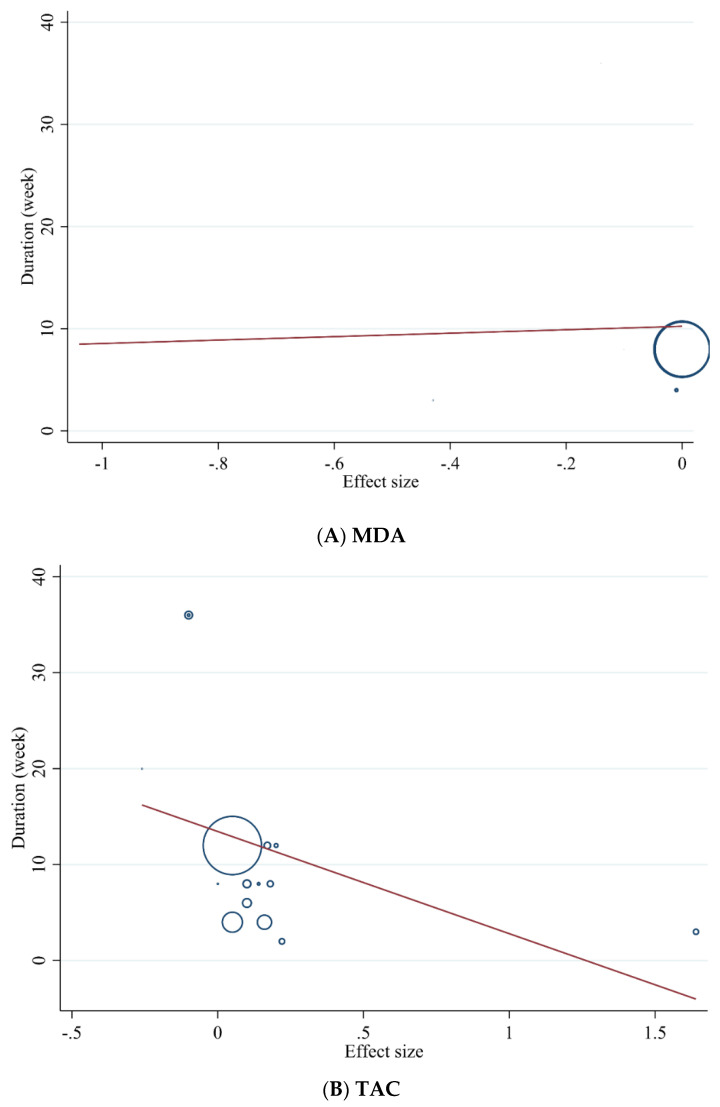
Random-effects meta-regression plots of the association between duration of intervention (weeks) and effect size of (**A**) MDA and (**B**) TAC.

We performed meta-regression analysis to evaluate the association between the dose of green tea (mg/day) and the duration of the intervention (weeks) with antioxidant status. The results of the analysis revealed that green tea supplementation significantly changed MDA (*r* = −2117.18, *p* = 0.017) based on the dose of the intervention (Figure 6) and did not show a linear relationship between dose and duration with significant changes in TAC (Figure 7 and Figure 8A,B). 

## 4. Discussion

To our knowledge, this is the first meta-analysis to evaluate the effects of green tea or green tea extract supplementation on oxidative stress (assessed TAC and circulating MDA concentrations). We identified 16 RCTs and 17 arms with 760 participants. These studies suggest that green tea and green tea extract supplementation were positively associated with TAC, but no association with MDA was observed in all subgroups. The association was independent of sex, BMI, duration, and green tea dosage. 

Increased oxidative stress elevates the risk of chronic diseases, such as cardiovascular and end-stage renal disease, as it is related to the onset and progression of these adverse conditions [11,12,62,63]. A greater antioxidant intake has been previously related to a lower risk of disease mortality [62]. In general, Camellia sinensis and its tea are known as one of the significant sources of antioxidants such as flavonoids. It has also been shown that green tea is especially rich in a very strong antioxidant called epigallocatechin gallate [63,64]. For the first time, our research demonstrated a significant beneficial effect of green tea supplementation on TAC via a systematic review and meta-analysis, indicating the decreasing impact of this compound on oxidative stress development. The existence of catechins such as EGCG and ECG in green tea, which have numerous free hydroxyl groups, have significant beneficial effects on free radical scavenging activity, and therefore, can lessen the product of lipid peroxidation [65]. A growing body of evidence has suggested the possible role of green tea supplementation in enhancing the antioxidant capacity [30,66,67,68,69]. Additionally, catechin improves antioxidant capacity and may result in a significant decrease in lipid peroxidation production, which has been supported by the study of Lin et al. [66].

Several possible mechanisms are responsible for green tea polyphenols on increased plasma TAC. The vigorous antioxidative function of catechins is related to the dihydroxyl or trihydroxyl groups on the B-ring and the meta-5,7-dihydroxyl groups on the A ring. This function can also be heightened by the trihydroxyl structure in the D-ring (gallate) in EGCG and epicatechin gallate. Based on the polyphenolic structure, free radicals can be neutralized by Electron delocalization [67]. Moreover, reactive oxygen and nitric radicals can be excluded by tea catechins [68,69].

The concentration of lipid peroxidation products representing oxidative stress across health and disease has not been adequately referenced [17,18,19,20]. In this present research, the MDA was proposed as an indicator of lipid peroxidation productions [70]. Regardless of the antioxidant activity of catechin, there are no consistent results on its effects on MDA. For instance, Hadi et al. in 2017 suggested that MDA concentrations were substantially subsided in athletes who consumed green tea extract [5]. This lowering effect of green tea on MDA was also confirmed by Noronha et al. 2019, which explains our findings [25]. Although we found that catechin has no significant effect on MDA concentrations in all subgroups, our meta-regression analysis demonstrated that green tea supplementation reduced MDA concentrations based on the dose of the intervention (mg/day).

On the contrary, several studies have shown no significant association between catechin and the value of MDA. The beneficial effects of green tea catechins in the elimination of ROS have been shown in vitro, although Heijnen et al. supported the idea that it may not be effective in vivo [71]. It has been observed that even with the highest dosage, the number of flavonoids and polyphenol in serum and intracellular are remarkably less than other types of antioxidant concentrations. It is also worth mentioning that some polyphenols, such as catechins, are the result of primitive polyphenols, and thus have poorer antioxidants levels than the parental polyphenols. It seems likely that dietary flavonoid actions as an antioxidant in vivo are not noticeable [72].

The present systematic review and meta-analysis is not without limitations. Most analyses had high levels of heterogeneity, but this was expected because included studies had different types of participants, doses, and intervention durations. In addition, there were not enough studies to evaluate all components of oxidative stress (MDA and TAC) in certain diseases. Moreover, our search was limited to RTCs published in English. Our meta-analytic work has several strengths, including RCTs and diverse participants (both healthy and diseased) from three different continents. Further well-designed RCTs are warranted to corroborate and expand on our outcomes, particularly in regions where green tea supplementation is not popular and in subjects with different diseases that may benefit from a decline in oxidative stress, such as those with cancer. 

## 5. Conclusions

In conclusion, combined data from interventional studies showed green tea supplementation has a beneficial effect on TAC. Additionally, a linear inverse association between the dose of green tea supplementation and a significant change in MDA was reported. Green tea and green tea extract can be proposed as suitable drinks to increase antioxidant status in adults, which may decrease the risk and progression of chronic diseases related to higher oxidative stress levels.

## Figures and Tables

**Table 1:** Characteristic of included studies in meta-analysis.

Author	Publication Year	Country	Study Design	Participant	Sex	Trial Duration (Week)	Means Age		Means BMI		Intervention			Sample Size	
							IG	CG	IG	CG	Treatment Group	Green Tea Dose (mg)	Control Group	IG	CG
Azizbeigi et al. 2009	2009	Iran	RCT	Obese men	M	8	23.9	22.8	31.8	30.8	Green tea extract	500 mg	500 mg sucrose	10	10
Jowko et al. 2011	2011	Poland	DB/R/PC	Healthy individuals	M	4	21.5	21.2	23.85	23.31	Green tea extract	640 mg of polyphenols	Maltodextrin	17	18
Bogdanski et al. 2012	2012	Poland	DB/R/PC	Obese, hypertensive patients	F/M	12	49.2	51.5	32.5	33.9	Green tea extract	379 mg	Pure microcrystalline cellulose	28	28
Suliburska et al. 2012	2012	Poland	DB/R/PC	Obese patients	F/M	12	48.56	52.26	32.07	33.45	Green tea extract	379 mg	Pure microcrystalline cellulose	23	23
Mousavi et al. 2013 (A)	2013	Iran	RCT	T2DM patients	F/M	8	54.6	52	27.4	28.1	Green tea	4 cup = 10,000 mg	-	26	14
Mousavi et al. 2013 (B)	2013	Iran	RCT	T2DM patients	F/M	8	56.2	52	28.1	28.1	Green tea	2 cup = 5000 mg	-	25	14
Lasaite et al. 2014 (A)	2014	Lithuania	DB/R/PC	T2DM patients	F/M	36	57.2	56.8	NR	NR	Green tea extract	400	Microcrystalline cellulose	17	14
Lasaite et al. 2014 (B)	2014	Lithuania	DB/R/PC	T2DM patients	F/M	36	57.2	56.8	NR	NR	Green tea extract	600	Microcrystalline cellulose	17	14
Spadiene et al. 2014 (A)	2014	Lithuania	DB/R/PC	T2DM patients	nr	36	62.18	62.18	35.23	34.98	Green tea extract	400	Microcrystalline cellulose	20	25
Spadiene et al. 2014 (B)	2014	Lithuania	DB/R/PC	T2DM patients	nr	36	62.18	62.18	35.23	34.98	Green tea extract	600	Microcrystalline cellulose	20	25
Mozaffari-Khosravi et al. 2014	2014	Iran	RCT	Type 2 Diabetes Mellitus	F/M	4	52.2	52.1	28	28.3	Green tea	450 mL	Sour tea	48	46
Kuo et al. 2015	2015	Taiwan	DB/PC	Healthy individuals	M	4	20	20	21.95	23.55	Green tea extract	250 mg	Starch capsule	10	10
Hadi et al. 2016	2016	Iran	DB/R/PC	Soccer players	M	3			22.6	22.82	Green tea extract	450 mg	450 mg of maltodextrin	18	18
Soeizi et al. 2017	2017	Iran	SB/R/PC	β–Thalassemia Major	F/M	8	23.1	24.2	20.9	19.42	Green tea	2500 mg	Warm water	26	26
Ribeiro Vaz et al. 2018	2018	Brazil	DB/R/PC	type 1 DM or type 2 DM	F/M	20	46.48	52.29	27.58	25.6	Green tea extract	1120 mg	cellulose capsules	27	28
Venkatakrishnan et al. 2018	2018	Taiwan	DB/R/PC	Hypercholesterolemic subjects	F	12	45	45	31.39	28.81	Green tea	600 mL (780.6 mg of catechin)	Tea flavor with very less concentration of catechin and caffeine	20	20
Sadowska-Krepa et al. 2019	2019	Poland	RCT	Male students of the physical education	M	6	22	23.1	23.73	24.72	Green tea extract	250 mg	Microcrystalline cellulose, magnesium stearate and maltodextrin	8	8
Sobhani et al. 2020	2020	Iran	RCT	Healthy individuals	M	2	26.12	24	23.85	24.62	Green tea extract	640 mg of polyphenols	Maltodextrin	8	7
Bazyar et al. 2020	2020	Iran	RCT	T2DM patients	F/M	8	51.75	52.61	29.46	29.28	Green tea extract	600 mg of EGCG	600 mg of wheat flour	22	22

Abbreviations: IG, intervention group; CG, control group; DB, double-blinded; SB, single-blinded; PC, placebo-controlled; CO, controlled; R, randomized; NR, not reported; F, female; M, male; RCT, randomized clinical trial.

**Table 2:** Quality assessment of the studies.

Study	Random Sequence Generation	Allocation Concealment	Blinding of Participants and Personnel	Blinding of Outcome Assessment	Incomplete Outcome Data	Selective Outcome Reporting	Other Sources of Bias
Mousavi et al. (2013)	U	H	H	U	L	L	U
Lasaite et al. (2014)	U	L	L	U	L	L	L
Spadiene et al. (2014)	U	L	L	U	L	L	L
Jówkoa et al. (2011)	U	L	L	U	U	L	U
Sadowska-Krępa et al. (2019)	U	L	H	U	L	L	U
Bogdanski et al. (2012)	U	L	L	U	L	L	L
Hadi et al. (2016)	U	L	L	U	L	L	L
Mozaffari-Khosravi et al. (2014)	U	H	U	U	U	L	U
Azizbeigi et al. (2019)	U	U	H	U	U	L	U
Ribeiro Vaza et al. (2018)	U	L	L	U	H	L	L
Suliburska et al. (2012)	L	L	L	L	L	L	L
Kuo et al. (2014)	U	L	L	L	L	L	L
Soeizi et al. (2017)	L	L	L	L	L	L	L
Venkatakrishnan et al. (2018)	U	L	L	U	L	L	L
Sobhani et al. (2020)	U	L	L	U	L	L	U
Bazyar et al. (2020)	U	L	L	U	L	L	L

Abbreviations: U; unclear risk of bias, L; low risk of bias, H; high risk of bias.

**Table 3:** Subgroup analyses of green tea supplementation on oxidative stress biomarkers.

	NO	WMD (95%CI)	*p*	P Heterogeneity	I^2^
Subgroup analyses of green tea supplementation on TAC concentrations.					
Overall effect	16	0.20 (0.09, 0.30)	<0.001	<0.001	98.6%
Trial duration (week)					
<8	5	0.43 (0.10, 0.75)	0.010	<0.001	99.6%
≥8	11	0.08 (0.02, 0.13)	0.004	<0.001	80.1%
Type of green tea					
Green tea extract	12	0.23 (0.03, 0.42)	0.019	<0.001	98.9%
Brewed green tea	4	0.07 (0.03, 0.10)_	<0.001	0.169	40.5%
Sex					
Both	8	0.12 (0.08, 0.17)	<0.001	0.173	32.0%
Male	6	0.38 (0.07, 0.69)	0.003	<0.001	99.5%
Female	1	0.05 (0.04, 0.05)	0.015	-	-
Intervention dose (mg/d)					
≤400	6	0.06 (−0.01, 0.13)	0.132	<0.001	87.1%
>400	10	0.29 (0.09, 0.49)	0.004	<0.001	99.1%
Health status					
Healthy	11	0.08 (0.02, 0.13)	0.004	<0.001	80.1%
Unhealthy	5	0.43 (0.10, 0.75)	0.010	<0.001	99.6%
Baseline BMI (kg/m^2^)					
18.5–24.9	6	0.38 (0.08, 0.68)	0.011	<0.001	99.5%
25–29.9	4	0.11 (0.07, 0.15)	<0.001	0.387	1.1%
≥30	5	0.06 (−0.03, 0.16)	0.176	<0.001	88.2%
Subgroup analyses of green tea supplementation on MDA concentrations					
Overall effect	8	−0.00 (−0.00, 0.00)	0.636	<0.001	84.1%
Trial duration (week)					
<8	3	−0.08 (−0.21, 0.04)	0.185	0.002	84.2%
≥8	5	−0.00 (−0.00, 0.00)	0.860	<0.001	86.3%
Type of green tea					
Green tea extract	4	−0.17 (−0.43, 0.09)	0.202	0.004	77.1%
Brewed green tea	4	−0.00 (−0.00, 0.00)	0.860	<0.001	89.5%
Sex					
Both	4	−0.00 (−0.00, 0.00)	0.860	<0.001	89.5%
Male	3	−0.18 (−0.51, 0.15)	0.499	0.002	84.3%
Intervention dose					
≤400	2	−0.01 (−0.02, 0.00)	0.215	0.530	0.0%
>400	6	−0.00 (−0.00, 0.00)	0.821	<0.001	88.1%
Health status					
Healthy	6	−0.00 (−0.00, 0.00)	0.853	<0.001	82.9%
Unhealthy	2	−0.20 (−0.61, 0.20)	0.331	<0.001	92.1%
BMI					
18.5–24.9	3	−0.46 (−0.98, 0.05)	0.081	<0.001	95.0%
25–29.9	3	−0.00 (−0.00, 0.00)	0.493	0.609	0.0%
≥30	2	−0.12 (−0.45, 0.20)	0.451	0.910	0.0%

Abbreviations: CI, confidence interval; WMD, weighted mean differences.
